# A rare epidermal growth factor receptor H773L/V774M compound mutation in advanced non‐small‐cell lung cancer with poor response to epidermal growth factor receptor tyrosine kinase inhibitor

**DOI:** 10.1002/rcr2.425

**Published:** 2019-04-10

**Authors:** Lun‐Che Chen, Jin‐Yuan Shih, Chong‐Jen Yu, Ching‐Yao Yang

**Affiliations:** ^1^ Division of Pulmonary and Critical Care Medicine, Department of Internal Medicine National Taiwan University Hospital Taipei Taiwan

**Keywords:** Afatinib, epidermal growth factor receptor, H773L/V774M, non‐small‐cell lung cancer, uncommon mutations

## Abstract

Uncommon mutations account for 10–15% of epidermal growth factor receptor (EGFR) mutations in patients with non‐small‐cell lung cancer. Afatinib is currently the most efficient EGFR‐tyrosine kinase inhibitor (TKI) against uncommon EGFR mutations. Here we report a 56‐year‐old woman presenting with persistent cough for one month. She was diagnosed with stage IV lung adenocarcinoma by bronchoscopic biopsy to the left lower lung tumour and serial image modalities. A rare H773L/V774M compound mutation in exon 20 was detected by gene sequencing. The patient received first‐line afatinib but primary resistance was noted with rapid left lower lung tumour progression. Second‐line chemotherapy combined with bevacizumab, pemetrexed, and cisplatin demonstrated more durable response. Our case suggests that H773L/V774M may be one of the EGFR‐TKI‐resistant uncommon EGFR mutations.

## Introduction

Mutation in epidermal growth factor receptor (EGFR) gene is one of the principal mechanisms leading to tumorigenesis of non‐small‐cell lung cancer (NSCLC), and was found in up to 50% of Asian, female patients who never smoked. In frame deletions of amino acids Leucine‐Arginine‐Glutamic acid‐Alanine (LREA) of exon 19 and exon 21 L858R point mutation are generally considered as “classical” or “common” mutations, which predict response to EGFR tyrosine kinase inhibitors (TKI) and account for approximately 85–90% of EGFR mutations. The remaining 10–15% of EGFR mutations are regarded as “non‐classical” or “uncommon” mutations and composed of a heterogeneous group of single or compound gene alterations within exons 18–21 1, 2.

Here, we report a case of advanced NSCLC with a rare EGFR exon 20 H773L/V774M compound mutation, which demonstrated poor clinical response to afatinib, a second‐generation EGFR‐TKI with pan‐human EGFR blocking activity. The anti‐cancer treatment was shifted to platinum‐based chemotherapy, which resulted in a partial response.

## Case Report

**Figure 1:**
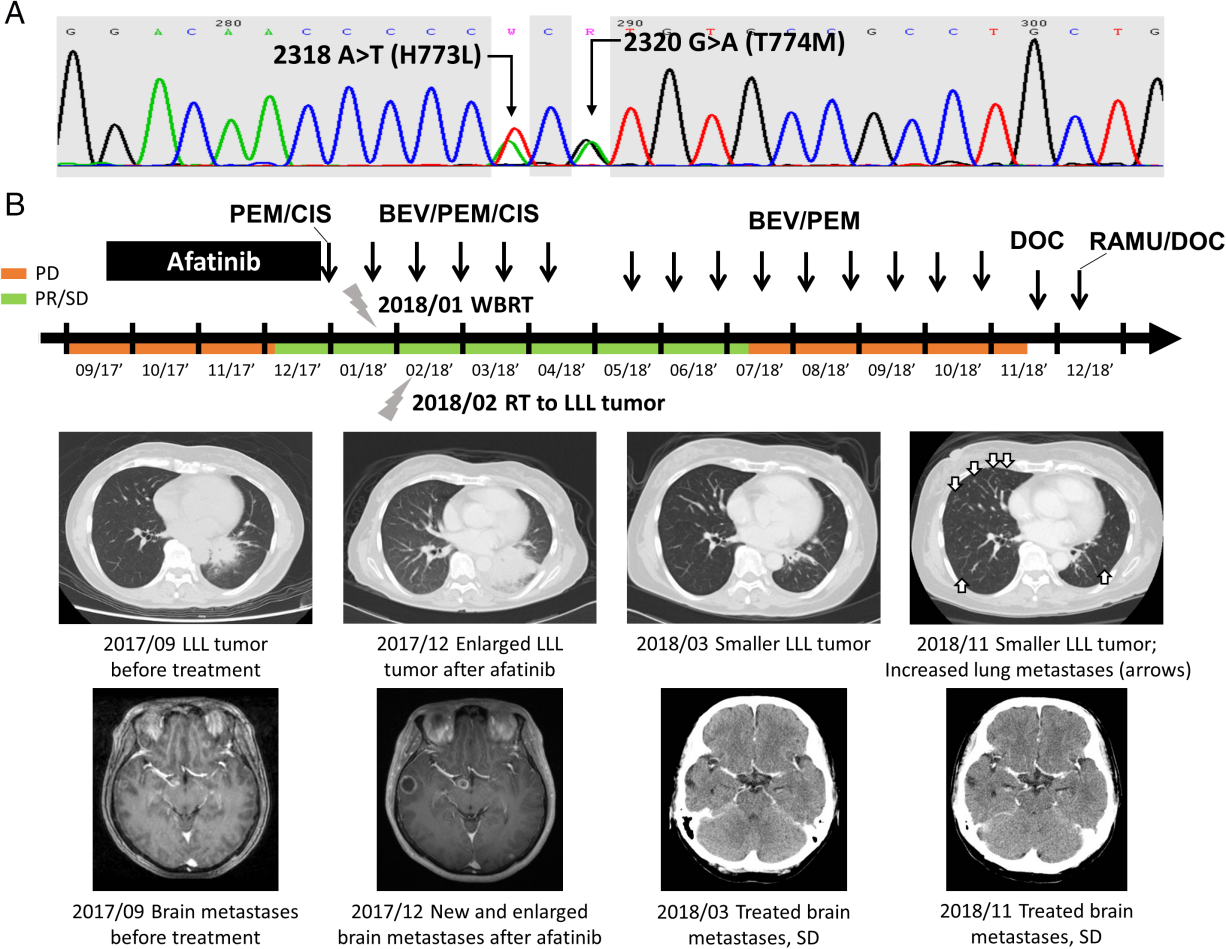
**(**A) Epidermal growth factor receptor c.2318A>T, p.2320G>A, p.H773V774>LM compound mutation in exon 20 detected by DNA sequencing. (B) Treatment course and clinical representative images of the patient. Abbreviations: BEV, bevacizumab; CIS, cisplatin; DOC, docetaxel; LLL, left lower lung; PD, progressive disease; PEM, pemetrexed; PR, partial response; SD, stable disease; RAMU, ramucirumab.

A 56‐year‐old never‐smoking female patient was diagnosed with stage IV lung adenocarcinoma (cT3N3M1b, according to American Joint Committee on Cancer, 8th edition) in September, 2017. Her tumour involved left lower lung (LLL), right supraclavicular, left infraclavicular to bilateral mediastinal lymph nodes, brain, T9 and L12 spine, and bilateral adrenal glands. A compound mutation in EGFR exon 20 (H773L/V774M complex) was found by Sanger sequencing of the tumour DNA extracted from paraffin‐embedded bronchoscopic biopsy specimens (Fig. 1A). The patient received first‐line afatinib (40 mg daily) but experienced rapid disease progression with enlargement of brain and lung tumours in first follow‐up three months later, indicating a pattern of primary resistance. Anti‐cancer therapy was shifted to pemetrexed (500 mg/m^2^ every three weeks, 15 cycles) and cisplatin (70 mg/m^2^ every three weeks, six cycles), with bevacizumab (7.5 mg/m^2^ every three weeks, 14 cycles) being added since the second cycle of chemotherapy. Sequential radiotherapies to whole brain (3300 cGy/10 fractions) and LLL tumour (3500 cGy/10 fractions) were also administrated. These managements led to a partial response until 10 months later while increased bilateral lung metastases developed. The therapies were then switched to a combination of ramucirumab (8 mg/kg every three weeks) and docetaxel (60 mg/m^2^ every three weeks), which resulted in a partial response after two cycles of treatments in the last follow‐up (November 2018). The treatment course was summarized in Figure 1B.

## Discussion

Non‐classical EGFR mutations are generally categorized into three groups: (i) exon 18–21 point mutations or duplications, (ii) de novo T790M, and (iii) exon 20 insertion, with various susceptibility profile to EGFR‐TKIs. Exon 20 insertions are generally considered resistant mutations 3, 4, with the exception of A763_Y764 insFQEA 5. G719X, S768I, and L861Q are the three most frequent non‐classical EGFR mutations other than exon 20 insertion, based on a pan‐Asian epidemiological study 6. Clinical researches evaluating patients with G719X, S768I, and L861Q mutations who received first‐line gefitinib or erlotinib demonstrated inferior treatment response rate, progression‐free survival, and even overall survival, compared with those with classical EGFR mutations 7. The second‐generation irreversible EGFR‐TKI, afatinib, exhibits more convincing efficacy against non‐classical EGFR mutations except exon 20 insertion. In pre‐clinical studies, the IC50 and IC90 ratio to classical EGFR mutations (i.e. L858R, exon 19 deletion) of gefitinib and erlotinib were much higher than that of afatinib 8, 9. Clinical studies focusing on the activity of afatinib against non‐classical EGFR mutations also showed compatible results (Table 1). The combined post‐hoc analysis of afatinib clinical trials (LUX‐Lung 2, LUX‐Lung 3, and LUX‐Lung 6) showed 71% overall response rate and 10.7 months progression‐free survival in patients with point mutations and duplications, or both, in exons 18–21 10.

**Table 1:** Clinical outcomes of non‐classical epidermal growth factor receptor mutations, treated with afatinib.

Article reference	Article type	Number of cases	Mutations*	ORR (%)	DCR (%)	PFS (months)	OS (months)
Yang et al. 10	Post‐hoc analysis of clinical trials (LUX‐Lung 2, 3, and 6)	75	(A) (*N* = 38)	71	84	10.7	19.4
			S768I (*N* = 8)	100	NA	14.7	NR
			G719X (*N* = 18)	78	NA	13.8	26.9
			L861Q (*N* = 16)	56	NA	8.2	17.1
			(B) (*N* = 14)	14	64	2.9	14.9
			(C) (*N* = 23)	9	65	2.7	9.2
Brueckl et al. 14	Prospective non‐interventional study	20	(A)	NA	NA	10.7	18.7
Wu et al. 15	Interim analysis of Phase IIIb, open‐label, single‐arm, multi‐centre study	35	(A)	NA	NA	9.1	NA
Shen et al. 16	Retrospective real‐world study	21	(A) + (B)	67	67	11.0	NA
			G719X/S768I/L861Q (*N* = 10)	70	70	18.3	NA
Kim et al. 17	Retrospective real‐world study	10	(A) + (C)	80	NA	NR	NA
Liang et al. 18	Retrospective real‐world study	22	(A) + (B) (exclude compound mutations with classical mutations)	NA	NA	11.5	NA

* Mutation groups: (A) exon 18–21 point mutations or duplications (including compound mutations with classical mutation(s)), (B) T790M, and (C) exon 20 insertion.

Abbreviations: DCR, disease control rate; NA, not applicable; NR, not reach; ORR, objective response rate; OS, overall survival; PFS, progression‐free survival.

Compound EGFR mutations, which comprised of two or more classical or non‐classical EGFR point mutations, is a small and unique group of non‐classical mutations with unknown clinical significance. Among them, concomitant point mutations at 2318A>T and 2320G>A leading to H773L and T774M amino acids alterations is an extremely rare non‐classical compound mutation. Upon a comprehensive literature review, only one retrospective study had reported a case with H773L/T774M mutations in 79 EGFR‐positive patients. A recent case report from China presented a patient with advanced NSCLC harbouring EGFR H773L/V774M compound mutations, who failed second‐line gefitinib but achieved sustained disease control to osimertinib 11. Structural analysis revealed this compound mutation leads to possible structural conflict between the α‐C helix and the loop after α‐C helix, pushing the C‐helix towards the active position and the binding pocket of EGFR‐TKIs. Similar to the pathogenesis of exon 20 insertion mostly occurs between the 770 and 774 residues, this presumed steric hindrance potentially results in drug resistance 12, 13.

After the US Food and Drug Administration broadened the indication of afatinib for metastatic NSCLC in 2018, it has become our EGFR‐TKI of choice when treating patient with non‐classical EGFR mutations. However, it is worth noting that there are still a minority of these mutations which do not have the same response with the non‐resistant non‐classical EGFR mutations (i.e. G719X, S768I, and L861Q). Our case showed that the rare EGFR compound mutation, H773L/V774M, had poor response to second‐generation TKI with irreversible pan‐human EGFR blockade activity.

### Disclosure Statement

Appropriate written informed consent was obtained for publication of this case report and accompanying images.
